# Effects of Resistance Exercise on Gastrointestinal Motility in Rats: Development of a Fluoroscopy-Based Experimental Model

**DOI:** 10.3390/jcm15218693

**Published:** 2026-11-07

**Authors:** Asseel Khalaf, Ajit Brindhaban, Hameed Al-Sarraf

**Affiliations:** 1 Radiologic Sciences Department, Faculty of Allied Health Sciences, Kuwait University, Kuwait City 13060, Kuwait; ajit.brindhaban@ku.edu.kw; 2 Physiology Department, Faculty of Medicine, Kuwait University, Kuwait City 13060, Kuwait; hameed.alsarraf@ku.edu.kw

**Keywords:** fluoroscopic imaging, gastric motility, resistance exercise, gastrointestinal physiology, preclinical imaging

## Abstract

**Introduction**: Exercise can alter gastrointestinal (GI) function, yet its effects on gastric function depend on the type, intensity, and duration of exercise. Despite extensive research on aerobic exercise, the gastric response to resistance exercise remains unexplored. This study aimed to develop a fluoroscopy-based model for quantifying gastric counts and to investigate changes in these measurements following resistance exercise in conscious rats. **Methods**: Adult male Sprague-Dawley rats were assigned to control and resistance-exercise groups (*n* = 8 per group). The exercise group underwent resistance training three times weekly for four weeks. Gastric counts were quantified in conscious animals using fluoroscopy immediately post-exercise, 24 h post-exercise, and at week 4, following oral administration of barium sulfate contrast medium (300 μL). Data are presented as mean ± SD. **Results**: Gastric counts were numerically lower in the exercise group than in controls immediately after exercise, at 24 h post-exercise, and following four weeks of resistance training. Between-group differences were not statistically significant at any assessment. A significant group × time point interaction was observed only at 24 h post-exercise (F = 3.06, *p* = 0.016). **Conclusions**: Resistance exercise was associated with time-dependent variations in gastric counts, with changes observed during the post-exercise period. These findings demonstrate the value of quantitative fluoroscopy for longitudinal assessment of gastric function and provide a framework for investigating the dynamic GI response to exercise in conscious preclinical models.

## 1. Introduction

Regular physical activity (PA) plays an important role in maintaining health and preventing disease [1,2,3,4]. The World Health Organization (WHO) defines it as any bodily movement produced by skeletal muscles that requires energy expenditure above resting levels [1]. Various health organizations recommend at least 150 min of moderate-intensity physical activity or 75 min of high-intensity activity each week, or an equivalent combination of both [5]. It has been suggested that PA can have either beneficial or harmful effects on the gastrointestinal (GI) tract, depending on the intensity, duration, and type of exercise [6]. High-intensity aerobic exercise is known to induce “Exercise-Induced Gastrointestinal Syndrome”; however, recent evidence suggests that resistance-trained adults also experience significant GI distress [7,8]. Research also suggests that regular exercise can help manage chronic GI conditions like irritable bowel syndrome (IBS) and gastroesophageal reflux disease (GERD) [9,10]. These improvements are likely linked to changes in gut microbial diversity and improved autonomic balance [11,12]. Animal models have further clarified these mechanisms, demonstrating that controlled exercise can inhibit tumorigenesis and accelerate healing in chemically induced colitis [13,14,15,16].

Additionally, PA may influence GI motility by affecting gastric emptying and intestinal transit time [17,18]. Exercise-induced GI disturbances are commonly reported, particularly during moderate-to-high-intensity activity [19]. They may manifest as symptoms such as heartburn, nausea, vomiting, abdominal discomfort, and, in severe cases, GI bleeding [20,21]. These effects are thought to arise from multiple physiological mechanisms. During exercise, blood flow is redistributed away from the splanchnic circulation toward working skeletal muscles, leading to transient intestinal ischemia and impaired GI function [22]. In addition, exercise has been shown to influence autonomic nervous system activity, gut hormone secretion, and neuroendocrine signalling pathways, all of which play key roles in regulating gastric motility and emptying. Together, these factors contribute to the complex and not yet fully understood relationship between physical activity and GI motility.

As PA may influence gastric emptying and intestinal transit time, imaging modalities are valuable for assessing these changes and improving our understanding of GI pathophysiology. Among these, magnetic resonance imaging (MRI) has emerged as a powerful, non-invasive tool for assessing gastric motility and emptying, offering high soft-tissue contrast, volumetric quantification, and the ability to perform repeated measurements in preclinical research [23]. However, MRI-based approaches in preclinical models often require anaesthesia, which can significantly alter GI motility and affect physiological interpretation [24,25]. Fluoroscopy, in contrast, is a powerful radiologic imaging modality that provides real-time visualization with superior temporal resolution and enables dynamic assessment across varying body positions [26]. Importantly, when combined with contrast media, fluoroscopy enables direct, in vivo visualization and quantitative assessment of GI motility in conscious animals, avoiding anaesthesia-related interference [26]. Using digital post-processing tools, fluoroscopy enables a precise quantitative assessment of structural and functional changes. In animal studies, fluoroscopy is a highly effective imaging tool for in vivo GI tract imaging [27,28,29]. Previous studies in rats have successfully used fluoroscopy to evaluate the effects of analgesics and antineoplastic agents on colonic motor function [30,31].

Despite extensive literature on aerobic activity, few studies have examined the potential role of resistance exercise in the GI tract. This study aimed to develop a fluoroscopy-based experimental model for quantifying gastric counts and to investigate the effects of resistance exercise on gastric function in an animal model, providing a foundation for future translational research.

## 2. Materials and Methods

### 2.1. Animal Model and Ethical Approval

Adult male Sprague-Dawley rats (10 weeks old) were obtained from the Animal Resources Center at Kuwait University. The rats were kept at an ambient temperature of ~23 °C under a 12-h light–dark cycle (lights on from 7:00 a.m. to 7:00 p.m.) and had free access to pelleted chow and water. Animals were divided into two groups (*n* = 8 each): a control group and an exercise group. The rats were treated in accordance with guidelines on the humane handling of experimental animals. Ethical approval was obtained from the Animal Resources Center Ethics Committee (AF-24-20).

### 2.2. Experimental Design

**Figure 1:**
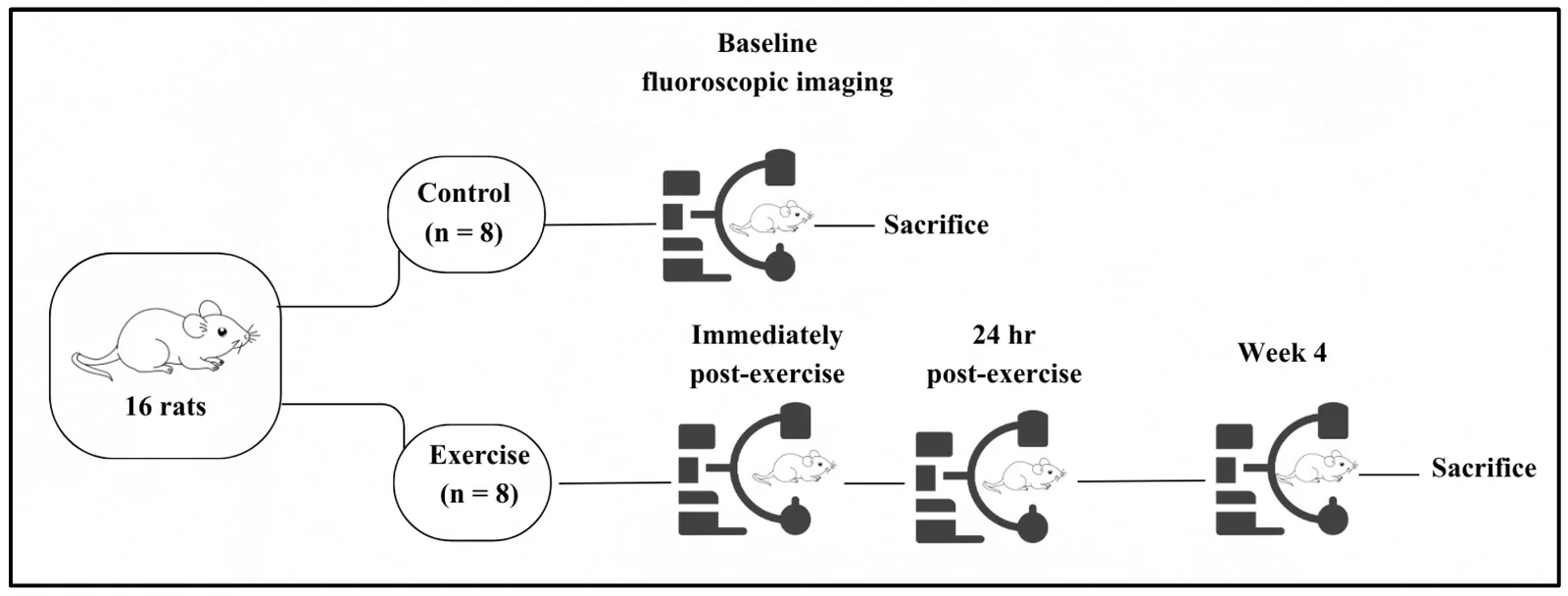
Timeline of the experimental protocol and fluoroscopic imaging assessments. Following a two-week conditioning period, control animals were assessed at baseline, while exercise animals were assessed immediately post-exercise, 24 h post-exercise, and at week 4.

Animals were divided into two groups (*n* = 8 each): a control group and an exercise group. Custom-built restraints were used to immobilize the rats during imaging. Rats were conditioned to the restraints for one-half hour per day for 2 weeks before imaging to reduce the stress and anxiety that might negatively affect the physiological parameters. Both groups underwent their initial fluoroscopic imaging assessment during week 1 of the experimental protocol, after which the control animals were sacrificed. The exercise group underwent subsequent imaging assessments immediately post-exercise, 24 h post-exercise, and at week 4, with the final assessment performed immediately following the last exercise session of the four-week resistance training programme (Figure 1). All imaging assessments were conducted at the same time of day to maintain consistency.

### 2.3. Resistance Exercise Apparatus

**Figure 2:**
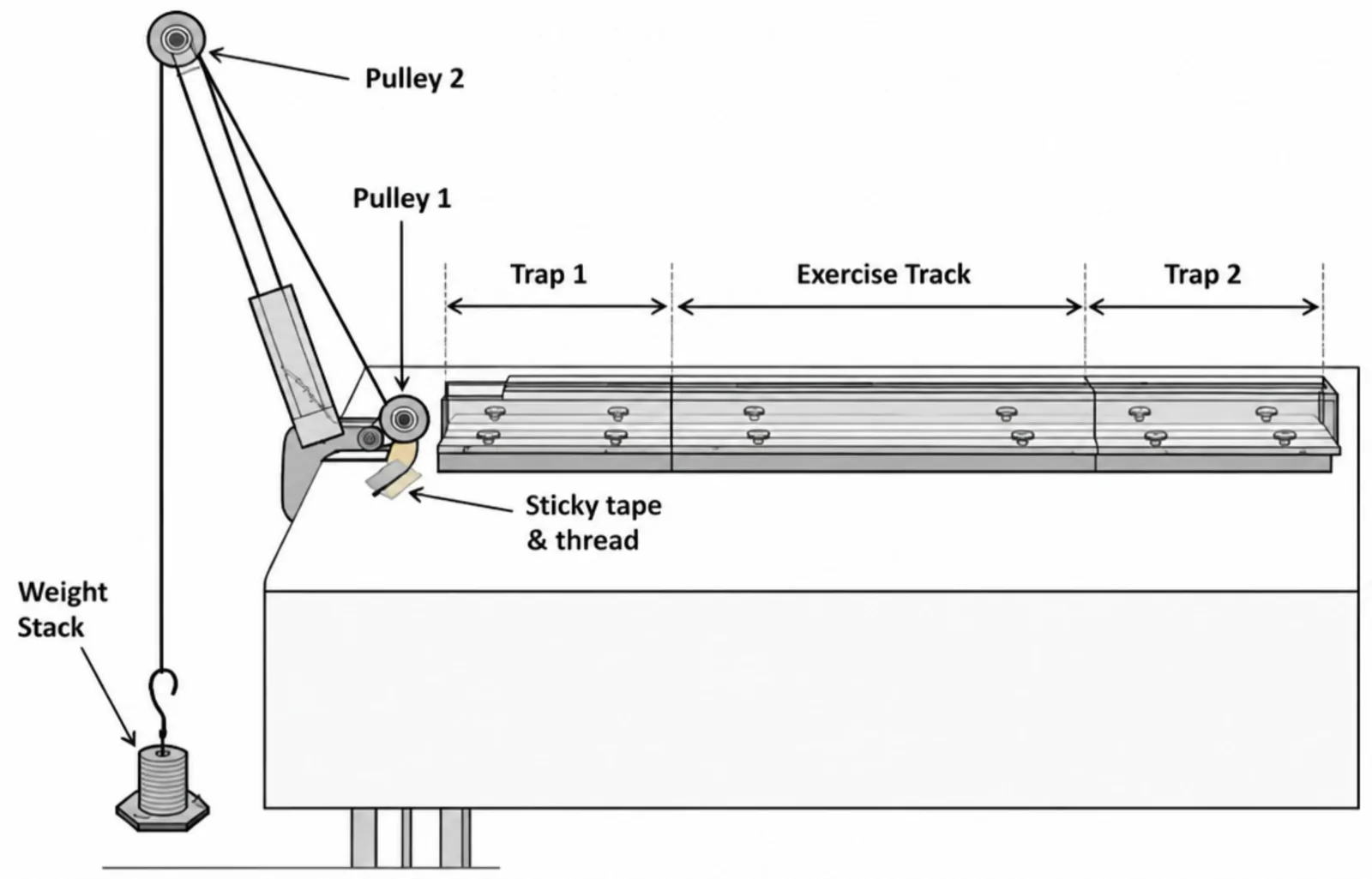
The complete rat resistance exercise system. The three-section transparent tunnel is positioned in front of a two-pulley tower. A strong cotton rope that runs over two pulleys is tied to a weight stack at one end and secured to the tail of a rat at the other end once the animal enters trap 1 [32].

The small animal resistance exercise apparatus used in this project consists of a three-section transparent Plexiglas tunnel with two identical sections (trap 1 and trap 2) at opposite ends of a longer middle section (exercise track). Altogether, these three sections form a continuous tunnel. This tunnel is placed on a table in front of a two-pulley tower (Figure 2) [32].

The exercise track is the main section where the animal pulls a predetermined weight attached to its tail. This section can be of three different sizes based on the type of resistance exercise required. At the start of the experiment, the rat was placed near the entrance of the trap 1 section. Once the rat entered this section, it was trapped by a closed sliding gate at the front of this section. A long thread, which is tied to a weight stack resting on the floor, is secured to the tail of the animal using sticky surgical tape.

### 2.4. Resistance Exercise Protocol

Rats were trained three times per week (Sundays, Tuesdays, and Thursdays) for four weeks. Each training session began with four warm-up runs carrying a load equivalent to 25% of body weight, followed by 7–9 runs with progressively increasing loads equivalent to 50%, 75%, and 100% of body weight, depending on each rat’s capability. The weights were increased gradually during the period according to the ability of the rat, as the goal of resistance training is to overload the muscle. The resting period between runs was 60 to 90 s.

### 2.5. Fluoroscopy Imaging System

A Radiologia ARCO FP C-arm fluoroscopy unit (Radiologia SA, Madrid, Spain) was used to record digital gray-scale images of the rats at a rate of up to 15 images per second. The digital images were analysed using Amira Visualization Software (Visage Imaging, Carlsbad, CA, USA).

### 2.6. Fluoroscopic Imaging Procedure

Rats were imaged four times, before the start of the resistance exercise, immediately after exercise, 24 h post-exercise, and at week 4 (Figure 1). Fluoroscopic imaging assessments were performed at the same time of day to maintain consistency across experimental time points. The final imaging assessment was conducted following completion of the four-week resistance training programme. Rats were conditioned to the restraints for one-half hour per day for 2 weeks before imaging to reduce the stress and anxiety that might negatively affect the physiological parameters. Liquid barium sulfate contrast medium (300 μL) was administered orally via a feeding tube into the stomach. Following oral administration of 300 μL of liquid barium sulfate contrast medium via a feeding tube, fluoroscopic image acquisition was initiated immediately to capture the early phase of gastric filling and subsequent emptying. Imaging parameters, including tube voltage, tube current, and frame rate, were standardized to maintain consistent exposure conditions across all imaging sessions. Standard radiation protection procedures were followed to ensure the safety of laboratory personnel.

### 2.7. Image Processing, Segmentation, and Quantification

Fluoroscopic image sequences were processed using Amira Visualization Software (Visage Imaging, Carlsbad, CA, USA). Image segmentation was performed using an intensity-based thresholding approach to isolate the barium contrast medium within the GI lumen. A threshold range corresponding to the grayscale intensity of the contrast medium was applied to generate segmented label images representing the distribution of the contrast agent. Segmented regions corresponding to the barium contrast were defined as regions of interest (ROI) and analysed using the Material Statistics module in Amira. The counts of grayscale intensities within each segmented ROI were extracted. This metric was used to quantify changes in gastric contrast distribution over time.

### 2.8. Statistical Analysis

Data are presented as mean values ± SD. Gastric counts were analysed using linear mixed-effects models, with group, imaging time points, and their interaction included as fixed effects. Rat identity was included as a random effect to account for repeated measurements within animals. Separate analyses were performed to compare the control group (*n* = 8) with the exercise group (*n* = 8) immediately post-exercise, at 24 h post-exercise, and following four weeks of resistance training. Bonferroni-adjusted pairwise comparisons were performed where appropriate. Values of *p* < 0.05 were considered statistically significant. Data analysis was performed using IBM SPSS Statistics ( version 31.0.1.0; IBM Corp., Armonk, NY, USA).

## 3. Results

### 3.1. General Parameters: Body Weight

**Figure 3:**
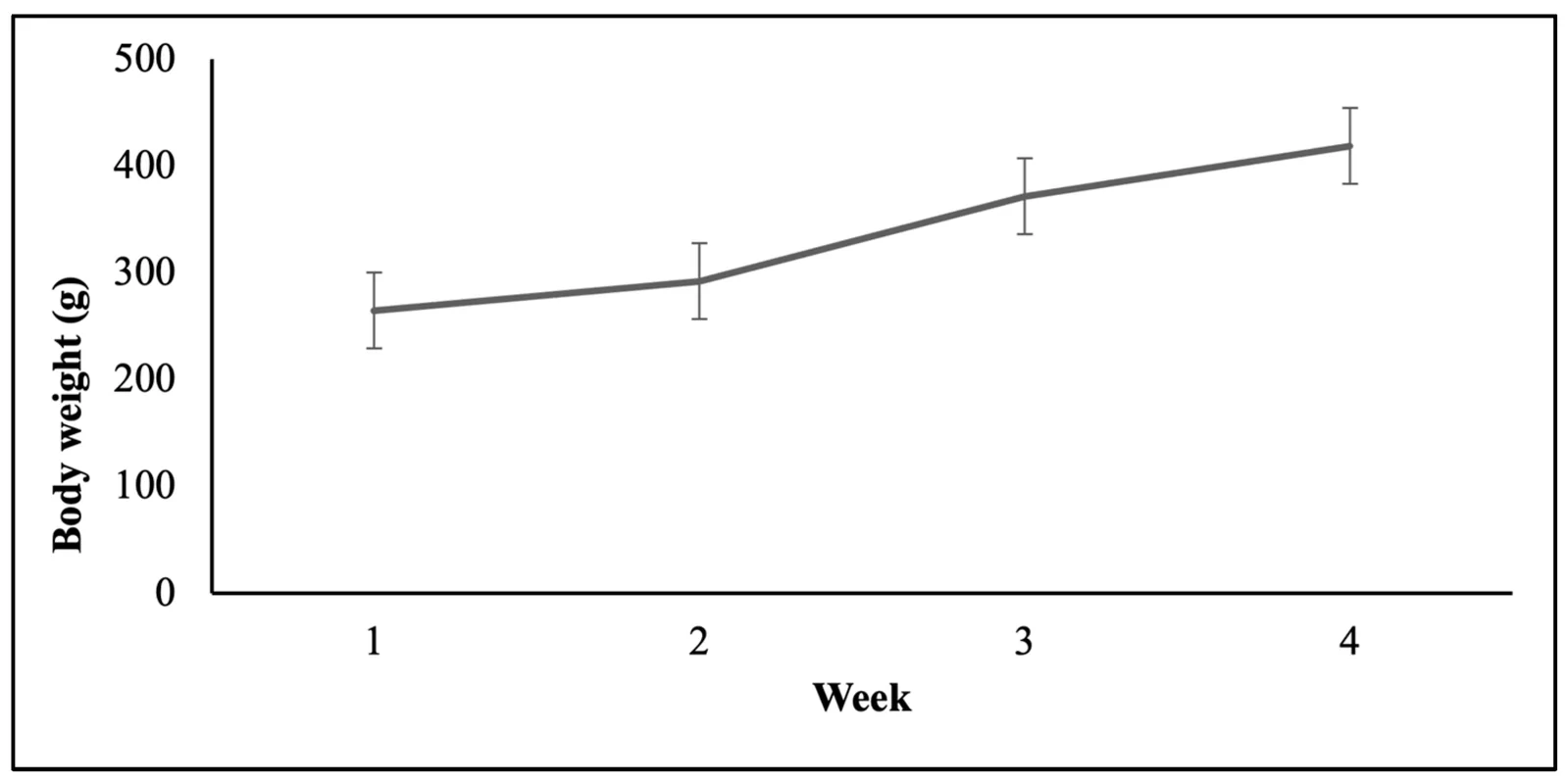
Body weight measurements in the exercise group (*n* = 8). Body weight was assessed weekly over the four-week experimental period. Data are presented as mean ± SD.

Animals’ body weight did not differ significantly between the control and exercise groups during the conditioning period (243.9 ± 34.3 g and 226.9 ± 6.8 g, respectively) or at week 1 (285.4 ± 57.7 g and 264.8 ± 12.9 g, respectively). In the exercise group, body weight gradually increased over the four-week resistance training period, from 264.8 ± 12.9 g at week 1 to 418.8 ± 11.8 g at week 4 (Figure 3).

### 3.2. Fluoroscopic Segmentation of Gastric Emptying

**Figure 4:**
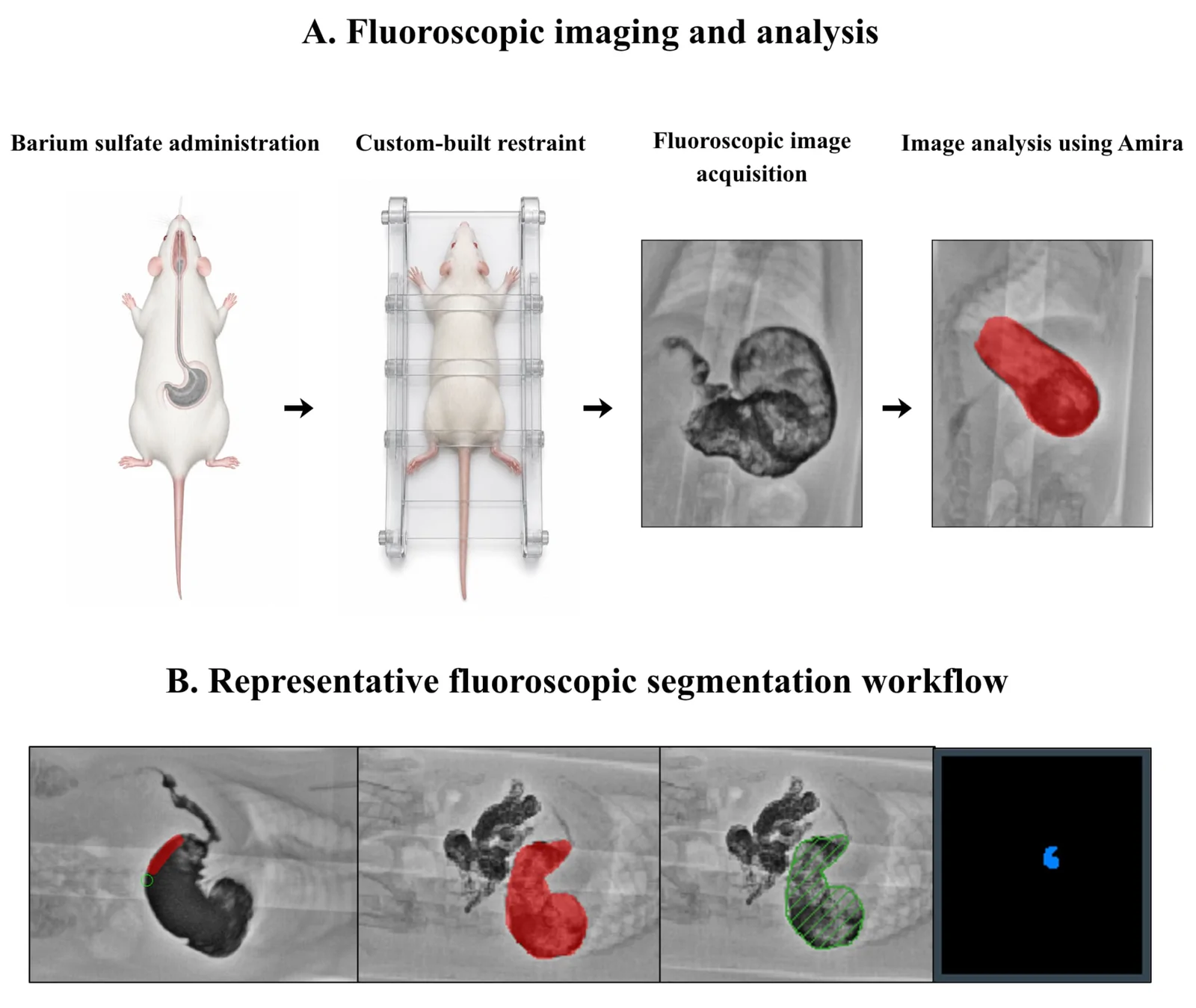
Fluoroscopic imaging and analysis workflow. (**A**) Experimental fluoroscopic imaging procedure showing the oral administration of barium sulfate, custom-built restraint, fluoroscopic image acquisition, and subsequent image analysis using Amira Visualization Software (Amira 3D Software 2024.2). (**B**) Representative screenshots illustrating segmentation of the contrast-filled stomach.

Fluoroscopic imaging enabled real-time visualization of gastric contrast distribution in conscious rats. Figure 4 shows representative fluoroscopic images and the corresponding segmentation of barium contrast generated using Amira. The segmented regions were used to quantify gastric counts (Figure 4).

### 3.3. Gastric Emptying Assessed by Fluoroscopy

**Figure 5:**
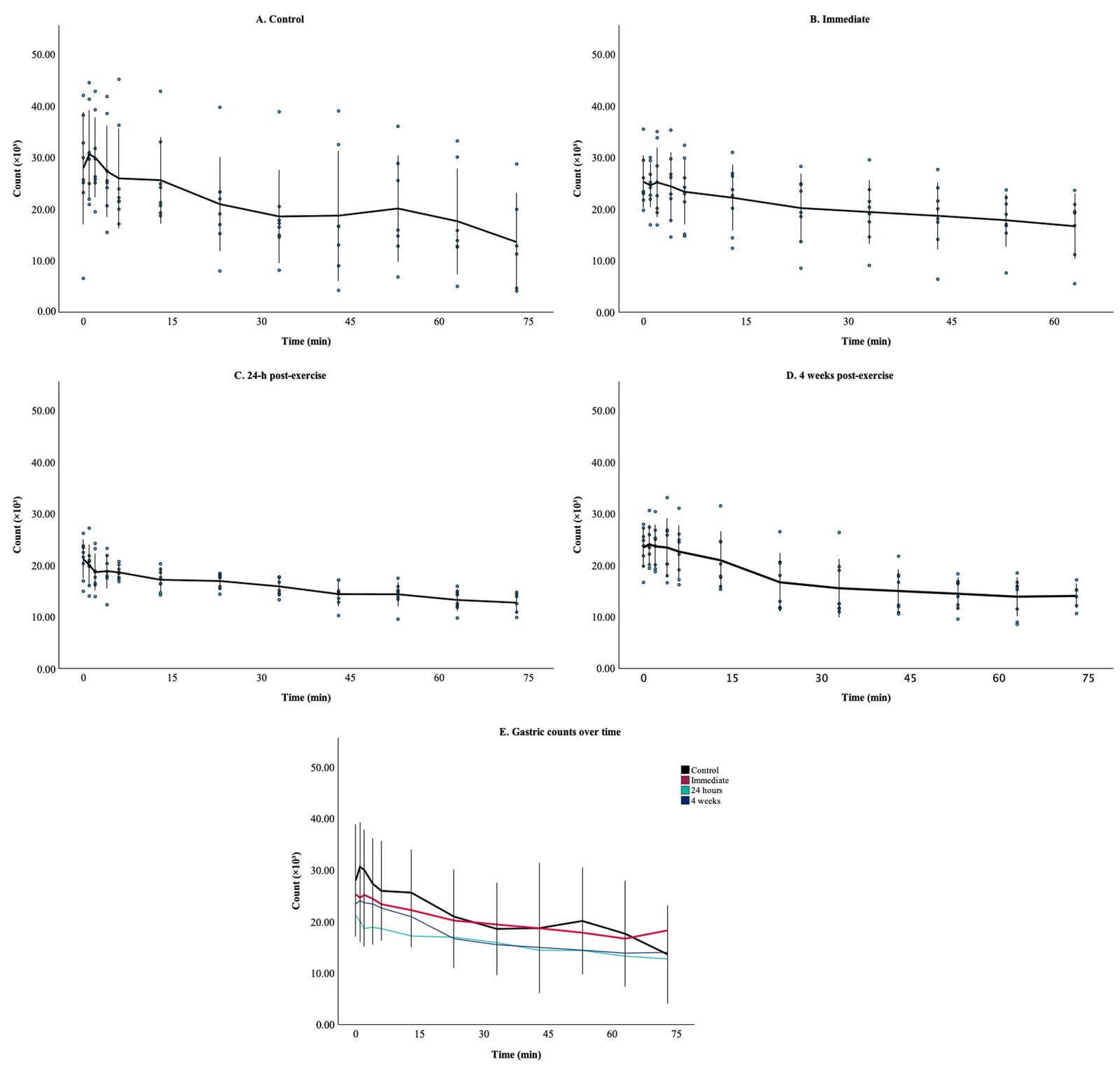
Gastric counts over time across experimental groups. Individual rats’ mean gastric counts are shown, measured at imaging time points in (**A**) control, (**B**) immediate, (**C**) 24 h, (**D**) week 4, and (**E**) combined comparison of all groups. The solid line represents the mean, and error bars represent SD. Data are presented as mean ± SD.

In the control group, gastric counts increased to a peak at 1 min, then declined progressively over the remainder of the recording period (Figure 5A). The immediate post-exercise group exhibited lower gastric counts than the control group from the initial time point and remained below the control values throughout the recording period (Figure 5B,E). At 24 h post-exercise, gastric counts followed a similar temporal profile and were lower than those observed in both the control and immediate post-exercise groups (Figure 5C,E). After 4 weeks, gastric counts increased relative to the immediate group but remained below the control values throughout the recording period (Figure 5D,E).

Linear mixed-model analysis revealed a significant main effect of time for the immediate (F = 20.14, *p* < 0.001), 24 h (F = 22.73, *p* < 0.001), and 4 weeks (F = 49.49, *p* < 0.001) post-exercise. A significant group × time point interaction was observed between the control group and the exercise group assessed 24 h post-exercise (F = 3.06, *p* = 0.016). Bonferroni-adjusted post hoc comparisons showed significant differences between the groups at time points 2 (*p* = 0.005), 3 (*p* = 0.003), 4 (*p* = 0.024), and 5 (*p* = 0.035).

**Figure 6:**
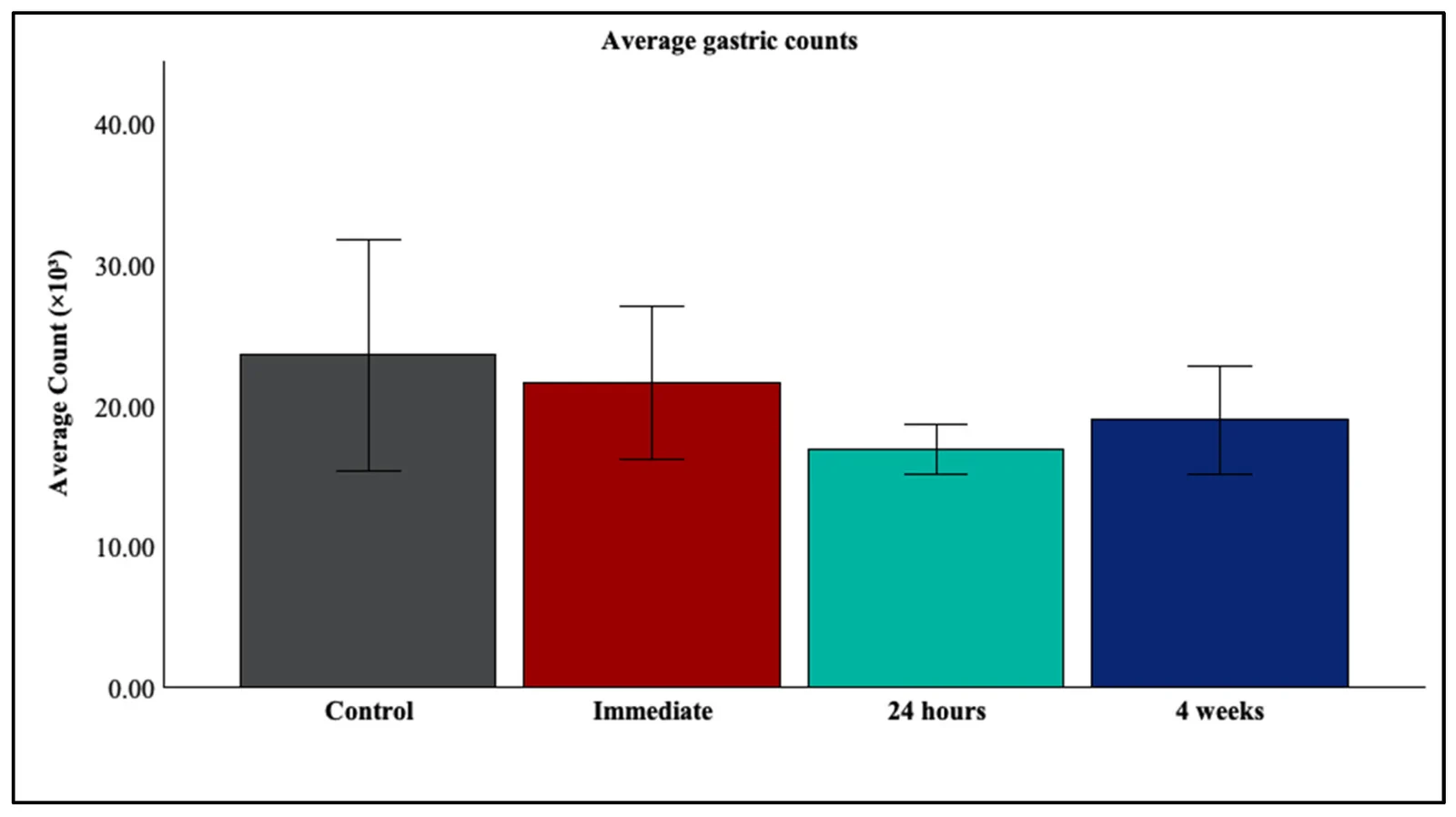
Average gastric counts across experimental assessment time points. Average gastric counts for the control group and the exercise group assessed immediately post-exercise, 24 h post-exercise, and at 4 weeks. Data are presented as mean ± SD.

No significant interaction between group and time was detected in any of the other comparisons. Mean gastric counts for each group are presented in Figure 6. Full fixed-effects results and pairwise comparisons are provided in Supplementary Tables S1 and S2.

## 4. Discussion

The present study investigated the effects of resistance exercise on GI motility using quantitative fluoroscopic imaging in a preclinical model involving conscious animals. The findings demonstrated significant changes in gastric counts over time following resistance exercise, with a significant group × time point interaction observed only at 24 h post-exercise.

Immediately following exercise, gastric counts were numerically lower than those observed under the control condition, although the difference was not statistically significant. At 24 h post-exercise, a reduction in gastric counts was observed, accompanied by a significant group × time point interaction, suggesting that the temporal pattern of gastric counts differed during the post-exercise recovery period. Following four weeks of resistance training, gastric counts increased relative to the immediate post-exercise measurements but remained numerically lower than control values throughout the recording period, although the difference was not statistically significant. Collectively, these observations suggest a time-dependent pattern of changes in gastric counts following resistance exercise. In the present study, a progressive resistance exercise protocol was employed rather than continuous aerobic or endurance exercise. Animals were trained three times per week for four weeks, with each session beginning with four warm-up runs using a load equivalent to 25% of body weight, followed by progressive loading at 50%, 75%, and 100% of body weight. Loads were subsequently increased according to the individual capacity of each animal, with 60–90 s recovery periods between runs. Previous animal studies indicate that the effects of exercise on gastric function may depend on the intensity and duration of exercise. Wang et al. [33] reported increased gastric antral contractile activity following mild exercise in rats, an effect that was abolished following vagotomy. In contrast, Cavalcante et al. [34] demonstrated that a single bout of higher-intensity swimming exercise delayed gastric emptying of a liquid meal compared with sedentary controls. Similarly, Silva et al. reported delayed gastric emptying following acute loaded swimming in awake rats [35]. The GI response to repeated resistance training remains less well characterized. Telles et al. [36] demonstrated that resistance training attenuated dexamethasone-induced delayed gastric emptying in rats, suggesting that prior exercise training may provide a protective effect against pharmacologically induced gastric dysmotility. These contrasting findings suggest that the gastric response to exercise may vary according to the type and modality of exercise, intensity, and duration, as well as the timing and method used to assess gastric function. This intensity-dependent response is also evident more broadly across the GI tract, as prolonged endurance and high-intensity exercise have been associated with adverse effects including GI bleeding, increased intestinal permeability, and alterations in the gut microbiota, with splanchnic hypoperfusion and mechanical stress among the proposed contributing mechanisms [37,38]. Collectively, these findings emphasize the importance of considering exercise intensity and modality when evaluating its effects on GI function, particularly when translating experimental findings into exercise recommendations.

A key outcome of this study was the successful development and implementation of a fluoroscopy-based experimental model for quantifying gastric counts. The model enabled objective assessment of gastric function across multiple time points, providing a practical approach for investigating temporal changes in GI function in an experimental setting. Fluoroscopy provides a minimally invasive approach for evaluating GI motor function in conscious experimental animals, avoiding the potentially confounding effects of anaesthesia and enabling dynamic assessment of intestinal contractility [28,30,31]. Previous MRI-based studies have provided quantitative assessment of gastric emptying and motility in rats; however, these measurements were predominantly performed under anaesthesia [24,25,39]. This approach enabled minimally invasive, longitudinal assessment of the movement and distribution of contrast material throughout the GI tract and allowed repeated evaluation at predefined time points following resistance exercise and training. The ability to visualize contrast progression through different GI regions provides an advantage for studying temporal changes within the same experimental framework. Furthermore, quantitative image analysis provided objective measures of contrast distribution and retention beyond visual fluoroscopic assessment alone.

The present findings may provide a basis for future investigations into the relationship between resistance exercise and GI function. Although resistance exercise is widely recognised for its musculoskeletal and metabolic benefits, its potential effects on GI function remain comparatively less characterised. The observed changes in gastric counts at 24 h post-exercise suggest that exercise-related differences may be detectable during the recovery period, although their physiological significance remains uncertain. Further preclinical and clinical studies incorporating direct measurements of gastric emptying and GI motility are needed to determine whether resistance exercise influences GI function. Future investigations incorporating physiological and biochemical measurements, together with assessments of skeletal muscle adaptations, may also help clarify the potential mechanisms involved.

Future studies should also investigate the potential contribution of the gut microbiota to GI responses following resistance exercise. Previous research has shown that resistance training can alter gut microbial diversity and composition in rats, suggesting a possible role for the gut microbiome in exercise-related GI changes [40]. Other exercise models have demonstrated variable effects on the gut microbiome, indicating that these responses may depend on the exercise paradigm [41,42]. Although the gut microbiome was not assessed in the present study, integrating microbiome analysis with longitudinal measurements of gastric counts and direct assessments of gastric motility may help clarify potential relationships between resistance exercise, microbial changes, and GI function.

Several additional limitations should be acknowledged. The present analysis was based on two-dimensional fluoroscopic images, which may result in overlap between GI structures and complicate the precise localization and quantification of contrast material. Although three-dimensional reconstruction may be achievable through post-processing, this was not performed in the present study. Quantitative measurements were also dependent on image segmentation and ROI definition, which may introduce measurement variability. The absence of complementary physiological and biochemical measurements further limits mechanistic interpretation. In addition, the limited number of assessment time points, particularly between 24 h and four weeks, prevents detailed characterization of the temporal evolution of the response and potential adaptation to repeated resistance training. Another limitation of the present study is the absence of a time-matched sedentary control group. Although the experimental design allowed gastric measurements to be obtained at different time points following exercise, control animals were assessed only at the initial time point. Consequently, potential influences of natural physiological growth, age-related changes in GI function, and stress associated with repeated experimental procedures cannot be fully excluded. The small number of animals per group and the absence of an a priori power analysis represent additional limitations of this study. However, given the exploratory nature of the study, the primary aim was to develop and establish a fluoroscopy-based experimental model for the quantitative assessment of gastric function.

## 5. Conclusions

In summary, the present findings suggest that the gastric response to resistance exercise may be time-dependent, with changes in gastric counts becoming more apparent during the post-exercise recovery period. Following four weeks of repeated resistance training, gastric counts remained numerically lower than control values, although the difference was not statistically significant. These findings highlight the importance of considering both exercise modality and the timing of GI assessment when evaluating exercise-induced changes in GI function. They also support the potential utility of quantitative fluoroscopic imaging for longitudinal evaluation of GI responses in preclinical models. Further studies incorporating additional recovery time points and complementary autonomic, metabolic, and GI motor assessments are needed to clarify the mechanisms underlying the responses and determine whether repeated resistance exercise induces adaptive changes in GI function.

## Data Availability

The datasets generated or analyzed during the study are available from the corresponding author upon reasonable request.

## Supplementary Materials

The following supporting information can be downloaded at https://www.mdpi.com/article/10.3390/jcm15218693/s1, Table S1. Summary of linear mixed-effects model analyses for group, time point, and their interaction; Table S2. Pairwise comparisons of gastric counts between control and exercise groups.
