# Supplementary material for: Effects of Resistance Exercise on Gastrointestinal Motility in Rats: Development of a Fluoroscopy-Based Experimental Model
Source: J Clin Med. 2026 Nov 7;15(21):8693. doi: 10.3390/jcm15218693 (PMC13764323; doi:10.3390/jcm15218693)
Supplement: Supplementary file 1 [file jcm-15-08693-s001.zip › Supplementary Table S1.pdf]

Supplementary Table S1: Summary of linear mixed-effects model analyses for group, time point, and their interaction.

Analysis	Effect	Numerator df	Denominator df	F	p-value
Immediate	Group	1	14.794	0.247	0.626
	Time point	11	14.694	20.141	<0.001
	Group × Time point	11	14.694	1.417	0.262
24 h	Group	1	14.401	4.231	0.058
	Time point	11	18.860	22.726	<0.001
	Group × Time point	11	18.860	3.055	0.016
4 weeks	Group	1	14.557	1.745	0.207
	Time point	11	20.542	49.486	<0.001
	Group × Time point	11	20.542	1.170	0.364

df: degrees of freedom.
p-values< 0.05 were considered statistically significant.
