# Supplementary material for: Effects of Resistance Exercise on Gastrointestinal Motility in Rats: Development of a Fluoroscopy-Based Experimental Model
Source: J Clin Med. 2026 Nov 7;15(21):8693. doi: 10.3390/jcm15218693 (PMC13764323; doi:10.3390/jcm15218693)
Supplement: Supplementary file 1 [file jcm-15-08693-s001.zip › Supplementary_Table S2.pdf]

Supplementary Table S2. Pairwise comparisons of gastric counts between control and exercise groups.

Assessment	Mean difference	95% CI	Adjusted p-value
Immediate	1,801.09	−5,925.97 to 9,528.15	0.626
24 h	6,365.59	−254.22 to 12,985.40	0.058
4 weeks	4,290.59	−2,650.44 to 11,231.61	0.207

Mean differences are expressed as control minus exercise. CI, confidence interval.
p-values< 0.05 were considered statistically significant.
